# Correlation of microRNA levels during hypoxia with predicted target mRNAs through genome-wide microarray analysis

**DOI:** 10.1186/1755-8794-2-15

**Published:** 2009-03-25

**Authors:** Jennifer S Guimbellot, Stephen W Erickson, Tapan Mehta, Hui Wen, Grier P Page, Eric J Sorscher, Jeong S Hong

**Affiliations:** 1 Departments of Genetics, University of Alabama at Birmingham, Birmingham, AL 35294, USA; 2 Gregory Fleming James Cystic Fibrosis Research Center, University of Alabama at Birmingham, Birmingham, AL 35294, USA; 3 Department of Biostatistics, University of Alabama at Birmingham, Birmingham, AL 35294, USA; 4 Department of Medicine, University of Alabama at Birmingham, Birmingham, AL 35294, USA; 5 Department of Cell Biology, University of Alabama at Birmingham, Birmingham, AL 35294, USA

## Abstract

**Background:**

Low levels of oxygen in tissues, seen in situations such as chronic lung disease, necrotic tumors, and high altitude exposures, initiate a signaling pathway that results in active transcription of genes possessing a hypoxia response element (HRE). The aim of this study was to investigate whether a change in miRNA expression following hypoxia could account for changes in the cellular transcriptome based on currently available miRNA target prediction tools.

**Methods:**

To identify changes induced by hypoxia, we conducted mRNA- and miRNA-array-based experiments in HT29 cells, and performed comparative analysis of the resulting data sets based on multiple target prediction algorithms. To date, few studies have investigated an environmental perturbation for effects on genome-wide miRNA levels, or their consequent influence on mRNA output.

**Results:**

Comparison of miRNAs with predicted mRNA targets indicated a lower level of concordance than expected. We did, however, find preliminary evidence of combinatorial regulation of mRNA expression by miRNA.

**Conclusion:**

Target prediction programs and expression profiling techniques do not yet adequately represent the complexity of miRNA-mediated gene repression, and new methods may be required to better elucidate these pathways. Our data suggest the physiologic impact of miRNAs on cellular transcription results from a multifaceted network of miRNA and mRNA relationships, working together in an interconnected system and in context of hundreds of RNA species. The methods described here for comparative analysis of cellular miRNA and mRNA will be useful for understanding genome wide regulatory responsiveness and refining miRNA predictive algorithms.

## Background

MicroRNAs (miRNA) are approximately 22-nucleotide, non-coding RNA sequences important in the control of gene expression. They are involved in a variety of cellular processes, including development, cell differentiation, signaling, and tumorigenesis[1], and are believed to represent 1% of the predicted genes in mammalian and nematode genomes[2,3]. Mammals in general (and primates in particular) appear to have a large number of miRNAs not found in other animal orders[2], suggesting that many functional miRNAs may have emerged during recent evolutionary periods. According to current functional and predictive models, each miRNA regulates multiple genes during differentiation and/or development at the transcription, translation, and posttranslational levels[1,4,5]. However, few of these targets and regulatory pathways have been experimentally validated, and the number of authentic (as opposed to predicted) miRNAs that exist in the mammalian genome as well as the actual number of their targets are not yet known.

Considerable effort has been directed toward understanding which mRNAs within the human genome are subject to regulation by miRNA-mediated repression. The *miRGen Targets *interface allows users to search either for targets or particular miRNA(s) that influence a particular gene. DIANA-microT[6], MiRanda[7], TargetScanS[5], and PicTar[4] are four genome-wide prediction algorithms whose results are available through miRGEN [8-11], an integrated database of (i) positional relationships between animal miRNAs and genomic annotation sets, and (ii) animal miRNA targets according to combinations of widely used prediction programs. These algorithms can provide quite a variable picture of miRNA behavior, and it is difficult to assess which *in silico *predictive method is best for identifying true miRNA targets[12]. It is probable that the use of multiple programs combined with mRNA expression profiling will be necessary to address this question. As a result, we considered four different algorithms (PicTar, TargetScanS, miRanda(microrna.org), and miRanda(miRBase)) in this report when assessing the relationship between mRNA and miRNA expression.

Previous studies have evaluated the influence of a particular miRNA on potential mRNA targets and found a high degree of correlation in specific tissues [13-15]. In general, these reports have examined the regulatory relationships between miRNAs and the genome wide transcriptome, with a focus on pathological conditions (such as cancer) rather than acute perturbations such as hypoxia, although hypoxia in particular has been shown to regulate discrete miRNAs [16-23]. Hypoxia results in a change in expression of a significant portion of the human transcriptome. After oxygen restriction, we observed down-regulation of hundreds of transcripts, including the cystic fibrosis transmembrane conductance regulator (CFTR), in which we could not identify a consensus hypoxia regulatory motif (HRE, hypoxia regulatory element[24]; an indicator of transcriptional regulation by hypoxia inducible factor (HIF)). In the present study, we hypothesized that many of these transcripts may be down-regulated by miRNAs.

Kulshreshtha et al[22] recently demonstrated a functional link between hypoxia and microRNA expression, although the relationship to mRNA expression was not evaluated. We therefore investigated the effects of hypoxia on a model epithelia and found that 3125 unique genes were significantly altered. Of these, approximately 53% were down-regulated, presenting 1649 unique possible targets for miRNA-mediated repression. Expression data from a miRNA Bioarray (see Methods) was compared with the mRNA expression profile, and the strength of correlation against predicted targets with differentially expressed miRNAs was analyzed using computational techniques we developed specifically for this purpose. We found no compelling evidence that miRNA-mediated repression plays a major role in down-regulation of CFTR, and present evidence that the individual miRNA levels do not correlate well with their algorithm-predicted target mRNAs. However, the groups of miRNAs predicted to regulate the same mRNA target were found to be co-regulated, indicating that a level of combinatorial control may exist.

## Methods

### Cell line and culture conditions

The HT29 (human colonic) cell line was obtained from ATCC  and seeded on 12-mm diameter Transwell filters (Corning-Costar, Corning, NY). Cells were cultured in media (HT29: McCoy's 5a medium supplemented with 7% Fetal Bovine Serum (FBS)) for 5–7 days (media bathing both the apical and basolateral compartments) at 37°C (5% CO_2 _– 95% air gas mixture). Under these conditions, cells form polarized monolayers with transepithelial resistances of >1000 Ω·cm^2^. In some monolayers, media was removed from the apical side to expose cells to air (A/L or air-liquid interface). Media remained on the apical surface (L/L or liquid-liquid interface) of other monolayers to a depth of one centimeter, a condition that markedly impairs access to ambient oxygen, conferring a hypoxic environment at the cell surface and a glycolytic cytosol due to an impairment of oxygen diffusion through liquid [25-27]. Several studies have established that altered physiology of cells under submerged conditions is primarily the result of a lack of oxygen (with otherwise equal volumes or composition of overlying media) [27-29]. For example, it has been determined that cells cultured under liquid (L/L) are hypoxic as judged by three criteria; (i) HIF protein level is increased in L/L culture, (ii) HRE-driven luciferase activity is increased, and (iii) well known genes activated under hypoxia such as VEGF are dramatically elevated. The assay represents a useful and commonly used test for observing changes *in vitro *due to hypoxia.

### mRNA expression array

Total RNA was purified from HT29 cells using the *mir*Vana™ miRNA Isolation Kit per manufacturer instructions (Ambion, Inc., Austin, TX), and RNA quality assessed before RNA labeling (2100 Bioanalyzer, Agilent, Palo Alto, CA). Detailed analysis procedures are presented in the Manufacturer's GeneChip Expression Technical Manual (Affymetrix, Santa Clara, CA). Briefly, 2 ug of total RNA from each sample was used to generate double strand cDNA by linear amplification using oligo dT-T7 primer and reverse transcriptase. Subsequently, biotin-labeled cRNA was synthesized by *in vitro *transcription (IVT) using 3'-Amplification Reagents for IVT labeling (Affymetrix, Santa Clara, CA) followed by cRNA fragmentation. The Affymetrix Human Genome U133 Plus 2.0 Array was used for hybridization. This array contains 54675 probes designed to over 47,000 transcripts and variants. Arrays were hybridized overnight at 45°C, and then washed, stained, and scanned on a GeneChip Scanner 3000 (Affymetrix, Inc., Santa Clara, CA). Gene expression levels were analyzed with GeneChip Operating Software (Affymetrix, Inc., Santa Clara, CA). Raw data were analyzed using Microarray Suite, Version 5.0 software (Affymetrix, Santa Clara, CA). The raw data set is available through Gene Expression Omnibus under accession number GSE9234.

### miRNA expression array

Total RNA was purified from HT29 cells using the *mir*Vana™ miRNA Isolation Kit per manufacturer instructions (Ambion, Inc., Austin, TX) which efficiently purifies RNA as small as 10 nucleotides. Expression profiling was then performed using the *mir*Vana miRNA Bioarrays V2 (Asuragen, Inc., Austin, Texas) which contains probes for all mouse, rat, and human miRNAs (266, 238, 482 confirmed miRNAs, respectively) in miRBase . Samples for microRNA profiling studies were processed by Asuragen, Inc. (Austin, TX) and the microRNA enriched fraction obtained by passing total RNA through a flash PAGE™ Fractionator apparatus (Ambion, Inc., Austin, TX) and cleaned. The 3' ends of RNA molecules were tailed and labeled using the *mir*Vana™ miRNA Labeling Kit (Ambion, Inc., Austin, TX). Amine-modified nucleotides were incorporated during the poly (A) polymerase mediated tailing reaction, and Cy3 succinimide esters (Amersham Biosciences (GE Healthcare), Piscataway, NJ) were conjugated to amine moieties on microRNAs. Hybridization to the *mir*Vana miRNA Bioarrays (Ambion, Inc., Austin, TX) was performed. The Cy3 fluorescence on the arrays was scanned at an excitation wavelength of 532 nm using a GenePix 4200AL scanner (Molecular Devices, Union City, CA). The fluorescent signal associated with the probes and local background was extracted using GenePix Pro (version 6.0, Molecular Devices, Union City, CA). The raw data set is available through Gene Expression Omnibus under accession number GSE9234.

### mRNA expression array analysis

The raw microarray data obtained from Microarray Suite v5.0 software were analyzed using a two-sided t-test corrected for unequal variances (Welch test) to compare the mean expression level for each gene between the two groups. A Bayesian posterior probability of being a false positive result (expressed as the false discovery rate, FDR) was estimated for each probe set individually, based on the Welch t-test p-values and using a mixture model[30,31]. We focused on the genes among those most differentially expressed that had corresponding probe sets with a lower than 1% FDR, that is, with a posterior probability of being differentially expressed of 99%. These genes were annotated with their chromosomal locations, UniGene number, LocusLink ID and Gene Ontology (GO) information (genes were grouped according to biological process, cellular component, or molecular function) using NetAffx resources[32]. We used Onto-Express (available at , last accessed in March 2007) [33] to calculate whether any of the GO terms were significantly over-represented among differentially expressed genes, as determined by a two-sided binomial test. The p-value calculation is only valid if the expression levels of the genes are independent, which is probably not the case in expression studies; thus, the p-values reported for these analyses should only be considered as heuristic ranking statistics. The fold change represents the ratio between microarray probe expression values.

### MiRNA expression array analysis

Thresholding and signal scaling were generated using algorithms selected by Asuragen. The background-adjusted fluorescent values generated by GenePix Pro were normalized for each microRNA using a variance stabilization method described by Huber et al[34], followed by a Welch two-sample t-test carried out for every gene; and a multiplicity correction was conducted to control FDR at 5% using a step-up approach, as described by Benjamini and Hochberg[35].

## Results

### Expression profiles of mRNA in HT29 cells

**Figure 1:**
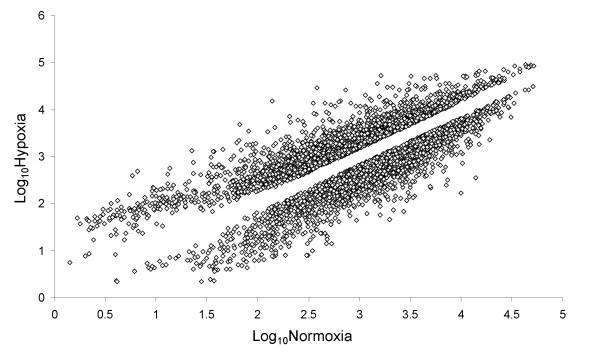
**Differentially expressed mRNAs**. Comparison of changes in HT29 mRNA levels under hypoxic vs. normoxic conditions. Unique genes that were differentially expressed 1.5 fold or greater are shown on the log-scale scatter plot. Upper cloud indicates transcripts at increased levels under hypoxic conditions and lower cloud indicates those decreased under hypoxic conditions.

Data analysis was performed using three replicates of array data per group (within group Mean r^2 ^= 0.996, range 0.004–0.998). Genes were considered differentially expressed between normoxic and hypoxic conditions if absolute expression changes were 1.5-fold or greater, a lower than 0.1% false discovery rate (FDR) was observed, and the p-value from a Welch two-sample t-test was less than 0.001. Over 8% of all probes satisfied these criteria (1999 probes up-regulated and 2099 down-regulated, see Figure 1). These probe sets map on to 1476 up- and 1649 down-regulated genes with a HUGO gene symbol, along with 208 probe sets that did not map to a HUGO identifier. Table 1 provides examples of selected genes known to be regulated by hypoxia with a broad biological significance, which are also predicted to contain target sites (in the 3'UTR) for miRNAs, according to the miRanda(microrna.org) target prediction database (for complete dataset of all genes altered by oxygen restriction in our studies, see GEO series GSE9234). Interestingly, the miRanda(mirorna.org) Target Database predicted that every 3'UTR in this set had target sites for between 4 and 69 miRNAs, although only 30% of the genome has been suggested to be regulated by miRNA-based mechanisms[1,4,5].

**Table 1:** Representative list of genes changed between hypoxia and normoxia including number of miRNAs predicted to have target sites in each gene (p ≤ 0.001).

**Symbol**	**Gene Description**	**Number of miRNAs as predicted by miRBase**	**Fold Change**
ALDOC	fructose-bisphosphate aldolase C	35	76.01
ANGPTL4	angiopoietin-like 4	69	74.01
PFKFB4	6-phosphofructo-2-kinase/fructose-2,6-biphosphatase 4	14	20.63
HIG2	hypoxia-inducible protein 2	34	16.55
EGLN3	egl nine homolog 3 (C. elegans)	13	13.80
CA9	carbonic anhydrase IX	15	12.04
VEGF	vascular endothelial growth factor	15	9.68

EGR1	Early growth response 1	15	0.02
DDX28	DEAD (Asp-Glu-Ala-Asp) box polypeptide 28	7	0.03
NOX1	NADPH oxidase 1	8	0.03
CFTR	cystic fibrosis transmembrane conductance regulator, ATP-binding cassette (sub-family C, member 7)	13	0.16
DDX52	DEAD (Asp-Glu-Ala-Asp) box polypeptide 52	6	0.18
DDX27	DEAD (Asp-Glu-Ala-Asp) box polypeptide 27	8	0.26
FPGT	fucose-1-phosphate guanylyltransferase	34	0.31

Examples of genes relevant to oxygen deprivation or related areas are shown.

### Over-represented GO Categories

We next analyzed our mRNA array data to identify various cellular processes affected by hypoxia according to Gene Ontology (GO) annotation[36]. GO is organized into three partially overlapping categories that consider three different aspects of each gene: biological processes, molecular functions, and cellular component. To investigate whether any GO terms were significantly over-represented among the differentially expressed genes, we used the Onto-Express tool[33] to calculate statistical significance values for each category. Categories with the most significant corrected p-value are shown in Table 2. The highest fraction of differentially expressed genes among the biological processes class included genes linked to cholesterol biosynthesis, protein metabolism, or ribosome biogenesis. Among the molecular function class, highly represented genes included those involved in L-ascorbic acid binding, ATP-dependent RNA helicase activity, oxidoreductase activity, NAD binding, and tRNA binding. A high fraction of mitochondrial inner membrane presequence translocase complex proteins was found among the cellular components class. Only a small portion of GO terms in each category changed significantly (less than 1% of entire terms, p < 0.05), suggesting the hypoxia-response may be highly specialized. Taken together, all three categories indicate considerable effects on glycolysis, translation and protein metabolism, and RNA processing. Of note was one group of proteins particularly affected by hypoxia. At least 20 members of the DEAD box family were significantly down-regulated, 2 to 33 fold, and comprised individual gene products in molecular function and biological process categories important in cellular pathways involving RNA (Table 3) [37,38]. These results suggest a substantial impact on RNA metabolism and activity by hypoxia.

**Table 2:** Most significant GO functions in three GO classes.

**Most significant GO Biological Processes**					
**GO Term**	**GO ID**	**Changed Genes**	**Total Genes in Class**	**P-Value**	**Fraction**

Cholesterol biosynthesis	GO:0006695	12	20	7.04E-09	0.600
Nuclear mRNA splicing, via spliceosome	GO:0000398	29	110	9.77E-09	0.264
glycolysis	GO:0006096	14	39	1.16E-07	0.359
Protein biosynthesis	GO:0006412	38	247	8.00E-07	0.154
transport	GO:0006810	50	415	8.94E-07	0.120
Protein metabolism	GO:0019538	9	17	4.90E-06	0.529
Protein folding	GO:0006457	28	174	5.59E-06	0.161
Ribosome biogenesis	GO:0007046	6	14	3.22E-05	0.429
metabolism	GO:0008152	39	316	9.36E-05	0.123
tRNA processing	GO:0008033	8	33	5.32E-04	0.242
Regulation of the cyclin dependent protein kinase activity	GO:0000079	8	34	9.72E-04	0.235
Amino acid biosynthesis	GO:0008652	6	22	1.01E-03	0.273
Lipid metabolism	GO:0006629	24	193	1.02E-03	0.124
Regulation of progression through cell cycle	GO:0000074	27	200	1.14E-03	0.135
Cell cycle	GO:0007049	41	338	1.64E-03	0.121
Regulation of translational initiation	GO:0006446	7	22	1.88E-03	0.318

**Most significant GO Molecular Function.**					

**GO Term**	**GO ID**	**Changed Genes**	**Total Genes in Class**	**P-Value**	**Fraction**

Nucleotide binding	GO:0000166	173	1405	6.92E-11	0.123
RNA binding	GO:0003723	78	389	4.22E-10	0.201
Oxidoreductase activity	GO:0016491	61	382	6.44E-10	0.160
ATP binding	GO:0005524	123	1116	1.20E-09	0.110
Unfolded protein binding	GO:0051082	28	143	2.68E-08	0.196
Protein binding	GO:0005515	220	2514	1.77E-07	0.088
Transferase activity	GO:0016740	101	958	1.86E-07	0.105
L-ascorbic acid binding	GO:0031418	6	11	1.65E-05	0.545
Lyase activity	GO:0016829	16	89	1.37E-04	0.180
binding	GO:0005488	47	399	2.37E-04	0.118
ATP-dependent RNA helicase activity	GO:0004004	7	18	4.61E-04	0.389
Isomerase activity	GO:0016853	15	90	5.54E-04	0.167
Ligase activity	GO:0016874	27	184	5.54E-04	0.147
Oxidoreductase activity	GO:0016702	6	18	6.72E-04	0.333
NAD binding	GO:0051287	8	24	7.05E-04	0.333
Catalytic activity	GO:0003824	27	192	7.40E-04	0.141
Transporter activity	GO:0005215	29	281	1.50E-03	0.103
Kinase activity	GO:0016301	22	163	1.65E-03	0.135
tRNA binding	GO:0000049	5	13	1.87E-03	0.385

**Most significant GO Cellular Component**					

**GO Term**	**GO ID**	**Changed genes**	**Total genes in class**	**P-value**	**Fraction**

cytoplasm	GO:0005737	114	895	5.00E-10	0.127
nucleus	GO:0005634	281	2999	5.00E-10	0.094
mitochondrion	GO:0005739	70	528	1.01E-09	0.133
endoplasmic reticulum	GO:0005783	49	405	2.47E-06	0.121
mitochondrial inner membrane presequence translocase complex	GO:0005744	5	11	2.82E-04	0.455
soluble fraction	GO:0005625	24	194	3.22E-04	0.124
nucleolus	GO:0005730	12	59	1.09E-03	0.203

The fraction reflects genes differentially expressed relative to the total number of genes in that class.

**Table 3:** DEAD and DEAH helicases down-regulated in response to hypoxia.

**Symbol**	**Gene description**	**Fold Change**
DDX1	DEAD (Asp-Glu-Ala-Asp) box polypeptide 1	0.51
DDX5	DEAD (Asp-Glu-Ala-Asp) box polypeptide 5	0.43
DDX10	DEAD (Asp-Glu-Ala-Asp) box polypeptide 10	0.26
DDX17	DEAD (Asp-Glu-Ala-Asp) box polypeptide 17	0.55
DDX18	DEAD (Asp-Glu-Ala-Asp) box polypeptide 18	0.31
DDX19A	DEAD (Asp-Glu-Ala-As) box polypeptide 19A	0.35
DDX21	DEAD (Asp-Glu-Ala-Asp) box polypeptide 21	0.29
DDX27	DEAD (Asp-Glu-Ala-Asp) box polypeptide 27	0.26
DDX28	DEAD (Asp-Glu-Ala-Asp) box polypeptide 28	0.03
DDX31	DEAD (Asp-Glu-Ala-Asp) box polypeptide 31	0.09
DDX39	DEAD (Asp-Glu-Ala-Asp) box polypeptide 39	0.34
DDX42	DEAD (Asp-Glu-Ala-Asp) box polypeptide 42	0.49
DDX52	DEAD (Asp-Glu-Ala-Asp) box polypeptide 52	0.18
DDX55	DEAD (Asp-Glu-Ala-Asp) box polypeptide 55	0.44
DDX56	DEAD (Asp-Glu-Ala-Asp) box polypeptide 56	0.28
DDX58	DEAD (Asp-Glu-Ala-Asp) box polypeptide 58	0.16
DHX29	DEAH (Asp-Glu-Ala-His) box polypeptide 29	0.5
DHX33	DEAH (Asp-Glu-Ala-His) box polypeptide 33	0.17
DHX36	DEAH (Asp-Glu-Ala-His) box polypeptide 36	0.51
DHX9	DEAH (Asp-Glu-Ala-His) box polypeptide 9	0.28

Listed are genes downregulated 2-fold or more (p ≤ 0.001) under hypoxia.

### miRNA expression profiles in HT29 cells

**Figure 2:**
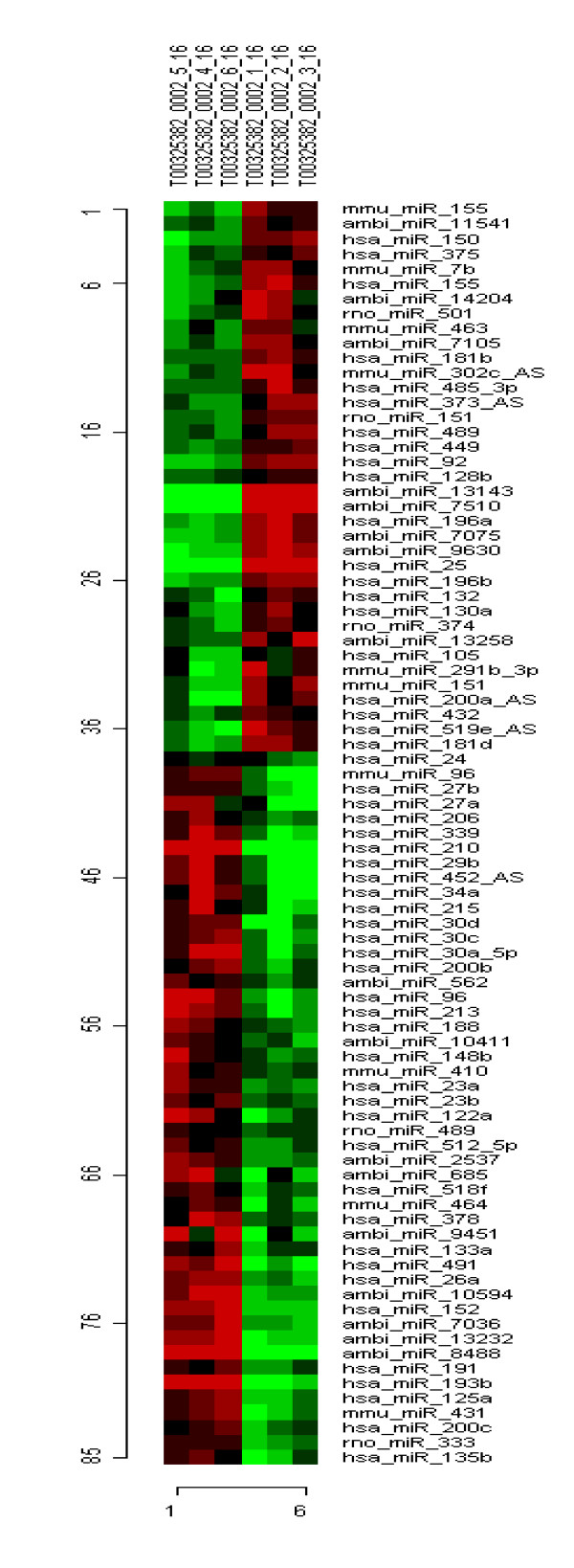
**Bicluster of microRNA expression**. Hierarchical clustering was carried out using correlation distance as the distance metric and average linkage between clusters to perform the analysis. This is a non-supervised method to illustrate potential relationships between the miRNA expression profiles from different samples. Hierarchical clustering was carried out for all samples and miRNA. The top of the figure indicates relationships between the various samples. The left-hand side shows the relationships between the miRNA identified on the right-hand side. The color of each cell reflects fold-change of the observed hybridization intensity relative to average hybridization intensity across all samples. Saturated green cells represent decrease in hybridization intensity, whereas saturated red cells represent an increase.

Unlike well-characterized hypoxia-mediated transcriptional activation[24], the general mechanisms underlying gene repression due to hypoxia are not well understood. We hypothesized that miRNAs may play an important role in the down-regulation of gene expression by hypoxia. We addressed this hypothesis by combining a comprehensive mRNA expression array with miRNA bioarray to evaluate not only the potential for a specific, hypoxic stress-induced miRNA profile, but also to correlate the expression of specific miRNAs with their algorithm-predicted targets. The *mir*Vana miRNA Bioarrays V2(Ambion, Inc., TX) used in this study contain probes for mouse, rat, and human miRNAs in miRBase. A hierarchical cluster analysis, using average linkage and Pearson's correlation as the weight function, of all the significant miRNAs evaluated in three hypoxic and three normoxic conditions is shown in Figure 2, illustrative of miRNAs differentially expressed by hypoxia. Approximately 8% (53 miRNAs) of all human miRNAs tested (640 unique miRNAs) were significantly different in the hypoxic group compared to the normoxic group. Twenty-eight miRNAs (4%) were up-regulated in a statistically significant fashion (p < 0.05) by hypoxia and twenty-five miRNAs (4%) were significantly down-regulated (p < 0.05) (Tables 4 and 5). No correlation of target mRNAs as predicted by miRanda (microrna.org) with any particular GO term was identified, supporting previous reports that miRNAs regulate a wide variety of mRNAs and their action is not restricted to specific groups of genes[5,39].

**Table 4:** MicroRNAs up-regulated by hypoxia (p ≤ 0.05).

	p-value
MicroRNA	(Hypoxia vs Normoxia)
ambi_miR_8488	0.00038
hsa_miR_152	0.00064
ambi_miR_13232	0.00137
hsa_miR_193b	0.00161
hsa_miR_26a	0.00204
ambi_miR_2537	0.00460
hsa_miR_512_5p	0.01019
mmu_miR_431	0.01033
hsa_miR_23a	0.01074
ambi_miR_7036	0.01075
hsa_miR_210	0.01103
hsa_miR_125a	0.01483
ambi_miR_10594	0.01708
hsa_miR_188	0.01969
hsa_miR_27b	0.02119
hsa_miR_191	0.02243
hsa_miR_30d	0.02262
hsa_miR_339	0.02339
hsa_miR_200b	0.02427
hsa_miR_23b	0.02885
hsa_miR_452_AS	0.03012
hsa_miR_491	0.03651
hsa_miR_30a_5p	0.03778
hsa_miR_30c	0.04013
rno_miR_333	0.04207
ambi_miR_562	0.04695
hsa_miR_213	0.04702
hsa_miR_206	0.04786

**Table 5:** MicroRNAs down-regulated by hypoxia (p ≤ 0.05).

	p-value
MicroRNA	(Hypoxia vs Normoxia)
hsa_miR_25	4.53630E-05
hsa_miR_196b	0.00014
ambi_miR_7510	0.00088
ambi_miR_13143	0.00129
ambi_miR_9630	0.00263
hsa_miR_92	0.00361
hsa_miR_181b	0.00507
hsa_miR_128b	0.00521
hsa_miR_196a	0.00894
hsa_miR_181d	0.00942
hsa_miR_449	0.00999
ambi_miR_7075	0.01053
rno_miR_151	0.01208
hsa_miR_150	0.01709
mmu_miR_7b	0.02328
hsa_miR_155	0.02363
hsa_miR_519e_AS	0.02637
mmu_miR_155	0.02638
ambi_miR_7105	0.02947
hsa_miR_373_AS	0.03220
mmu_miR_302c_AS	0.03309
hsa_miR_200a_AS	0.03337
hsa_miR_489	0.04173
ambi_miR_11541	0.04190
mmu_miR_151	0.04840

### Correlation of differentially expressed genes with the expression of their predicted regulatory miRNAs

**Figure 3:**
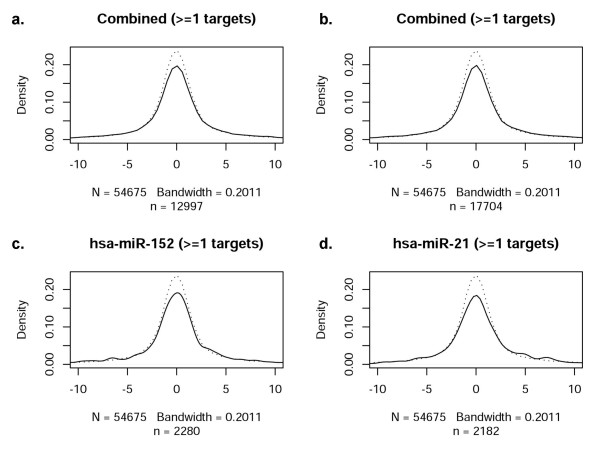
**Correlation of miRNAs with at least one target site in miRanda-predicted target mRNA**. In each panel, the reference distribution of t-statistics from all probes of the mRNA expression arrays is given by a dotted line. These are compared with the distribution of t-statistics for those probes that are predicted targets of given miRNAs, shown as a solid line. 'N' represents the number of transcripts in the reference sample (dotted line), and therefore is the same in each plot. Sample size (n) is the number of genes predicted to have target(s) of given microRNA(s) (solid line), and therefore changes from plot to plot. Note that *n *depends on the number of predicted target probes contained within the dataset, either combined among all miRNAs, or specific to an individual miRNA. The plotted distributions are Gaussian kernel density estimates (loosely, smoothed histograms), and the indicated bandwidth is in terms of the standard deviation of the smoothing kernel. The *x*-axis in each figure reflects the *t*-statistics for the comparison between groups (dotted line represents random distribution). The *y*-axis represents the density of observations at a given *t*-statistic value. a. Combined *t*-statistics for all significantly changed miRNAs with 1 site in the 3'UTR of predicted target miRNAs. b. Combined *t*-statistics for a set of non-significant miRNAs with 1 predicted target site. c. Representative significantly changed miRNA compared to all of its predicted targets. d. Representative non-significantly changed miRNA compared to all of its predicted targets.

**Figure 4:**
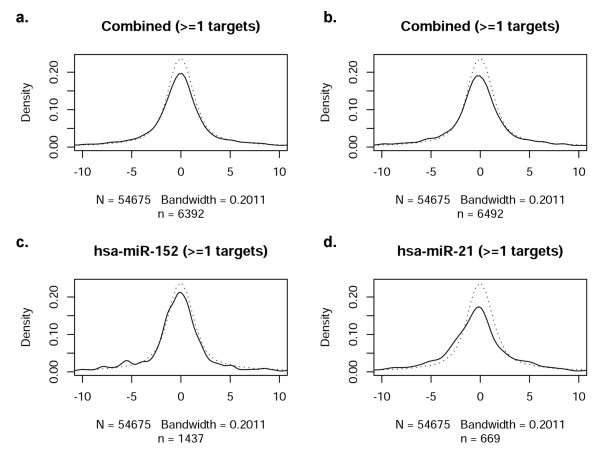
**Correlation of miRNAs with at least one target site in PicTar-predicted target mRNA**. a. Combined t-statistics for all significantly changed miRNAs with 1 site in the 3'UTR of predicted target mRNAs. b. Combined t-statistics for a set of non-significant miRNAs with 1 predicted target site. c. Representative significantly changed miRNA compared to all of its predicted targets. d. Representative non-significantly changed miRNA compared to all of its predicted targets.

**Figure 5:**
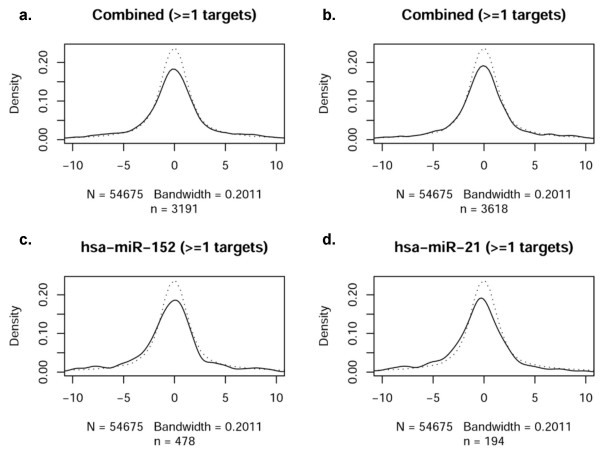
**Correlation of miRNAs with at least one target site in TargetScanS (with total context score)-predicted target mRNA**. a. Combined t-statistics for significantly changed miRNAs (top 15%) with 1 site in the 3'UTR of predicted target mRNAs. b. Combined t-statistics for a set of non-significant miRNAs with 1 predicted target site. c. Representative significantly changed miRNA compared to top 15% of its predicted targets. d. Representative non-significantly changed miRNA compared to top 15% of its predicted targets.

**Figure 6:**
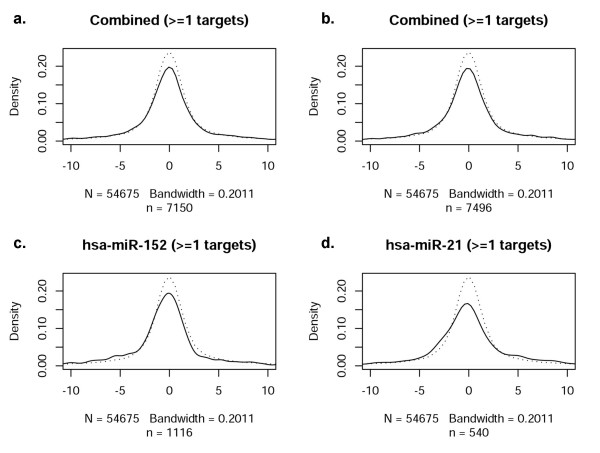
**Correlation of miRNAs with at least one target site in miRanda(miRBase)-predicted target mRNA**. a. Combined t-statistics for all significantly changed miRNAs with 1 site in the 3'UTR of predicted target mRNAs. b. Combined t-statistics for a set of non-significant miRNAs with 1 predicted target site. c. Representative significantly changed miRNA compared to all of its predicted targets. d. Representative non-significantly changed miRNA compared to all of its predicted targets.

While it is well established that miRNAs play an important role governing gene expression, the correlation of mRNA and miRNA profiles across the entire genome and in response to an acute cellular perturbation (such as hypoxia) have not been thoroughly assessed. Accordingly, we next evaluated the extent to which the profile of miRNAs observed during hypoxia was consonant with mRNA expression (i.e., the transcriptome response). The distribution of t-statistics for all probes on mRNA expression arrays was used as a reference distribution (dotted line), as shown in Figure 3. In comparison to the reference distribution, the thirteen most significant differentially expressed miRNAs (Tables 4 and 5) and twenty randomly selected non-significant miRNAs were analyzed. The distribution of t-statistics for mRNA probes that are predicted targets of the given miRNAs were generated (solid lines, Figures 3a and 3b). Figure 3 depicts the distribution of mRNAs with one predicted target site for a given miRNA (microrna.org); a deviation from the reference curve (dotted lines) would be interpreted as a significant relationship between the mRNA and miRNA arrays, as mediated via the target predictions. This methodological approach to investigating 'cause and effect' allows a visualization of mRNA/miRNA correlations. No consistent deviations, however, were observed in the combined t-statistics. As a further test of this finding, evaluations of individual miRNAs were performed (representative examples in Figures 3c and 3d). No consistent deviations were noted in t-statistics for any single miRNA in either the significant or the non-significant miRNA group. Figure 3 illustrates the distribution for miRanda-predicted targets, while Figure 4 through Figure 6 contain the results for PicTar, TargetScanS, and miRBase, respectively. When all miRNAs that changed significantly during oxygen restriction were compared against mRNAs containing one target prediction site in the 3'UTR, none of the target prediction programs indicated a significant relationship between data from the mRNA and miRNA expression arrays. When the findings were evaluated using the most recent TargetScanS predictive method (i.e. a more stringent threshold [40] that includes only the highest ranked targets with a context score percentile of 85%), no consistent deviations were observed in the combined t-statistics (Figure 5).

**Figure 7:**
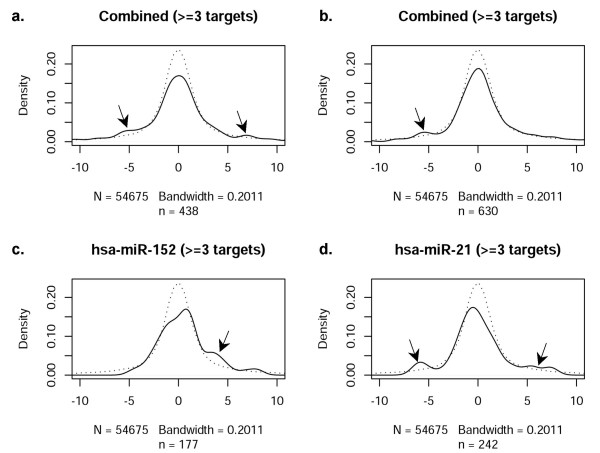
**Correlation of miRNAs with at least 3 target sites in miRanda-predicted target mRNA**. Same as **Figure 3**, except that probes identified as targets of a miRNA are required to have at least *three *target sites in the 3'UTR region according to the miRanda(microrna.org) target prediction software. Arrows indicate deviation from the reference graph.

Similar comparisons were made for mRNAs containing at least three target sites within each 3'UTR for the same miRNA (Figure 7). As in Figure 3, the 13 most significant differentially expressed miRNAs (Figure 7a) and 20 randomly selected, non-significant miRNAs (Figure 7b) were investigated. Certain miRNAs did exhibit some relationship with their predicted targets (indicated by arrows); however, miRNAs without statistically significant differential expression *also *demonstrated a roughly comparable relationship (arrows, Figures 7c and 7d), suggesting that this observation occurred by chance.

**Figure 8:**
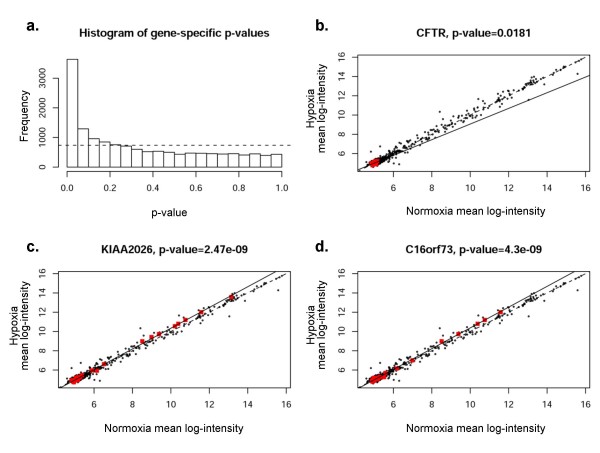
**Correlation of specific genes with predicted groups of miRNAs (miRanda, microrna.org)**. The expression levels in hypoxia and normoxia of each gene specific miRNA group were plotted. Significant deviation of the regression line (solid) from the line of equality (dotted line) indicates co-regulation of the group. **Panel a **shows histogram of the *p*-values for miRNA groups of all coding genes represented in the study for miRanda. Frequency on *y*-axis refers to the number of genes involved. **Panels b-d **depict CFTR, KIAA2026, and C16orf73 as examples of mRNA regulation by gene-specific miRNA groups. The red dots indicate gene-specific miRNAs for each given gene; this includes 9 miRNAs predicted by miRanda(microrna.org) for CFTR, 28 each for KIAA2026 and C16orf73.

Combinatorial regulation by groups of diverse miRNA species binding to different numbers of target sites within a single 3' UTR of a given gene has been hypothesized as a mechanism underlying miRNA-mediated gene repression[4]. Therefore, we next compared expression under hypoxia or normoxia of all miRNAs predicted to target a specific gene (termed a gene-specific miRNA group, Figure 8). This comparison evaluates whether specific groups of miRNAs (namely those predicted to target a specific gene) are significantly up- or down-regulated as a cluster. For each gene, a regression line (solid line, estimated best fit using the specific group of miRNAs indicated by red dots) was plotted and an ANOVA test performed to determine whether the regression line differed significantly from equality (dotted line). Figure 8a shows a histogram of the resulting p-values, indicating that a higher-than-expected number (expected result shown by dotted line) of gene-specific miRNA groups were regulated coordinately in association with a particular mRNA target.

**Figure 9:**
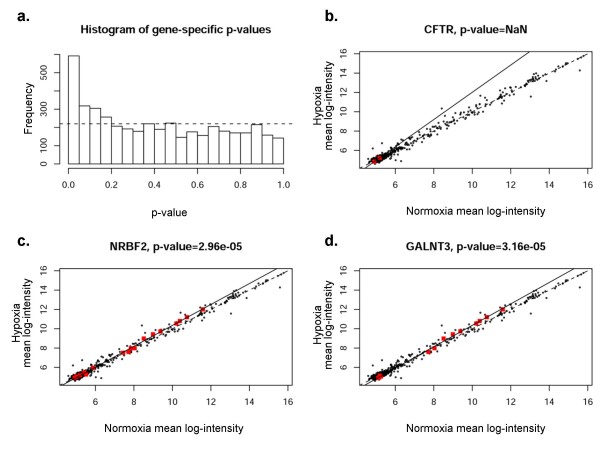
**Correlation of specific genes with predicted groups of miRNAs (PicTar)**. The expression levels in hypoxia and normoxia of each gene specific miRNA group were plotted. Significant deviation of the regression line (solid) from the line of equality (dotted line) indicates co-regulation of the group. **Panel a **shows histogram of the p-values for miRNA groups of all coding genes represented in the study for PicTar. Frequency on y-axis refers to the number of genes involved. **Panels b-d **depict CFTR, NRBF2, and GALNT3 as examples of mRNA regulation by gene-specific miRNA groups. The red dots indicate gene specific miRNAs for each given gene.

**Figure 10:**
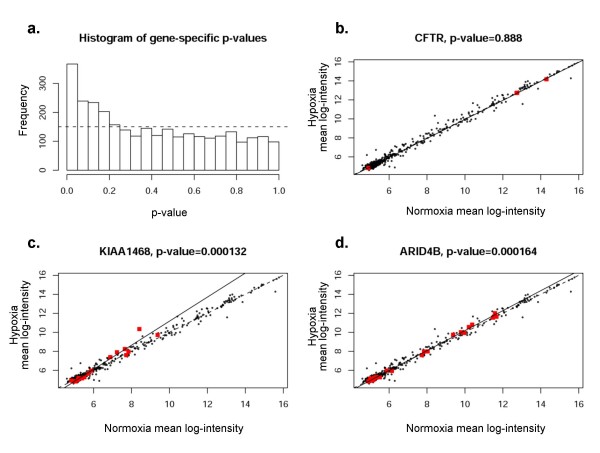
**Correlation of specific genes with predicted groups of miRNAs (TargetScanS)**. **Panel a **shows histogram of the p-values for miRNA groups of all coding genes represented in the study for TargetScanS. Frequency on y-axis refers to the number of genes involved. **Panels b-d **depict CFTR, KIAA1468, and ARID4B as examples of mRNA regulation by gene-specific miRNA groups. The red dots indicate gene specific miRNAs for each given gene.

**Figure 11:**
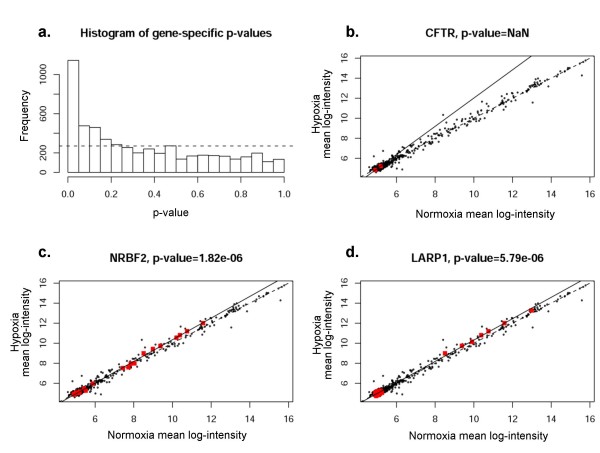
**Correlation of specific genes with predicted groups of miRNAs (miRanda/miRBase)**. **Panel a **shows histogram of the p-values for miRNA groups of all coding genes represented in the study for miRanda/miRBase. Frequency on y-axis refers to the number of genes involved. **Panels b-d **depict CFTR, NRBF2, and LARP1 as examples of mRNA regulation by gene-specific miRNA groups. The red dots indicate gene specific miRNAs for each given gene.

A representative sample of statistically significant gene-specific miRNA groups is shown in Figures 8b–d. We chose to analyze three genes; CFTR, KIAA2026, and C16orf73. Selection of CFTR was based on our laboratory interest in regulation of that particular gene product. The other two genes shown in the figure (whose functions are not known) were selected because of highly significant p-values, indicating very strong co-regulation by gene-specific miRNA groups. Scatterplots were identical by this method (black dots representing all miRNAs), indicating the group of miRNAs predicted to regulate expression of a particular gene of interest. Significant deviation from the line of equality (dotted line) indicates co-regulation of a cohort of miRNAs. The results presented here therefore provide some of the first evidence to suggest that miRNAs may be coordinately regulated in groups relevant to specific 3' UTRs. For example, Figure 8b shows predicted regulation of CFTR mRNA expression by CFTR specific miRNAs (red dots, 9 predicted by miRanda(microrna.org)). The regression line indicates considerable deviation, suggesting that coordinated regulation of CFTR mRNA may exist by these specific miRNAs. CFTR mRNA levels were among the most significantly decreased under hypoxia based on the mRNA array (Table 1). Figure 8 shows results for miRanda-predicted targets. Results for PicTar, TargetScanS, and miRanda(miRBase) are provided in the Figures 9, 10, 11. All four algorithms indicate gene-specific groups of miRNAs reacted coordinately to hypoxia, as shown by the histograms of p-values. However, different algorithms identified somewhat different gene-specific groups as being most strongly affected by hypoxia, as shown by the scatterplots (Figures 8b–d; and Figures 9, 10, 11).

Several mRNA changes were statistically significant due to pronounced differential expression of a single miRNA (for example Figure 10), a finding that may or may not be biologically relevant. In addition, while many mRNAs within a gene-specific miRNA group were found to be coordinately up-regulated, many others predicted to have target sites by miRanda(microrna.org) and other algorithms were not differentially expressed (Figure 8), suggesting that the definition of gene-specific miRNAs requires further refinement. Only a subset of genes with significantly regulated gene-specific miRNA groups were differentially expressed in our data set. The implication of these findings is that although miRNAs may be coordinately regulated, they do not predict expression changes of every predicted target.

## Discussion

Cellular responses to hypoxia can occur through stabilization of HIF, a well-established transcriptional activator, and result in enhanced expression of a variety of hypoxia-related genes. Much less is known regarding hypoxia-dependent transcriptional repression. Our mRNA array data indicate that a large number of transcripts are robustly downregulated following oxygen restriction in human epithelial cells. One goal of the present study was to investigate the extent to which changes in miRNA could account for these variations in the cellular transcriptome. We hypothesized that miRNAs would play a role suppressing certain genes during hypoxia, and tested this by comparing expression data from miRNA and mRNA expression profiles investigated in parallel.

Recent studies describe hypoxia specific miRNA signatures in a variety of cell types [16-23]. A functional link between hypoxia and miRNA expression has therefore been observed by others, although the relationship between mRNA and miRNA from a genome-wide perspective has not been investigated previously. Kulshreshtha and colleagues emphasized that a spectrum of miRNAs can be induced during hypoxia, and at least some of these occur via a HIF-dependent mechanism. Ten miRNAs reported previously as hypoxia-responsive were also identified in our experiments (e.g. miR-23a, -23b, -27b, -30d, -191, -210, -213, -155, -200a, -181b) using a different method of oxygen deprivation (Tables 4 and 5). Interestingly, three miRNAs (miR-155, -200a, -181b) reported to be upregulated in other cell systems (reviewed in [16-23]) were noted to be repressed in colonic epithelia. These differences most likely relate to the various cell-types, growth conditions, or procedural aspects used in earlier studies.

The epithelial model of hypoxia described here represents a well-defined *in vitro *system for studying subacute (including transcriptional) effects of oxygen restriction [25-29]. We used the model to evaluate miRNA regulation of gene expression. We found changes in the epithelial transcriptome resulting from low oxygen, as well as further evidence for a potential signature of miRNAs induced by hypoxia [16-23]. However, in contrast to several extant models [13-15], we did not observe a significant correlation between mRNA expression levels and miRNAs on a genome-wide scale. Earlier studies have relied primarily on particular tissue types and developmental stages from a variety of organisms, suggesting results most relevant to embryologic gene regulation. The present investigation of mRNA:miRNA association applied a novel analytical approach to widely available data visualization tools, and monitored miRNA and mRNA expression on a genome-wide basis, including the potential role of environmental stressors (found commonly in pathologic conditions) on miRNA-mediated regulation.

Our analysis incorporated four miRNA target predictions programs (MiRanda, PicTar, miRBase, TargetScan). When miRNA targets were compared to mRNA output, the data sets failed to indicate a significant relationship between expression arrays. In a very recent study, Baek et al. [40] reported that the top third TargetScan predictions (ranked by 'total context score') may correlate best with protein downregulation. In the present experiments, applying this stringent threshold and strict site conservation (after [40]) did not result in a stronger association. This included use of the most recent TargetScan algorithm (release 4.2; ) and restriction of targets to a context score of 85% or higher. Our results therefore indicate limitations of the currently available target prediction algorithms. While high stringency methods can be valuable for an individual miRNA [40], TargetScan/PicTar modifications do not appear to enhance the available algorithms in a broader, genomic context.

The lack of a significant and robust correspondence between mRNA levels and miRNA expression could represent a lack of specificity and/or accuracy of miRanda or other target prediction algorithms. The observed magnitude of miRNA expression changes (Figures 8b–d) in the present experiments is lower than observed for mRNA (Figure 1). In addition, the relatively small sample sizes used in this study could contribute to a lack of information, making it difficult to test the assumptions underlying the statistical method (such as normality), in a fashion that could impact results. MiRanda typically produces more potential targets than other programs, and a large number of false targets would seriously limit the computational methods described here. We also note that the available programs have only partially overlapping predicted targets for the same miRNA and produce smaller data sets than miRanda. Due to the differences among databases and because there are no clearly superior methods, future studies of mRNA and miRNA regulation should consider analysis of multiple predictive algorithms rather than use of a single data analysis tool.

Although miRNAs can act to promote cleavage and subsequent degradation of their mRNA targets, this may not be the only (or even primary) mechanism of miRNA action in mammalian epithelia. A strong consensus is not yet available regarding the predominant pathway(s) that underly miRNA gene repression [41-44]. One explanation for our findings could relate to translational repression as a major action of miRNA in human cells. It has been shown that certain miRNAs bind their targets and prevent adequate translation. However, mRNA levels are not always affected by this process. A quantitative, proteomic approach to evaluate hypoxic protein expression in epithelia followed by *in silico *statistical correlation would be necessary to investigate this possibility. On the other hand, miRNA levels are also governed by DNA promoter elements, stability of miRNA, degradative pathways related to differential RNA editing, transport into the cytoplasm, and/or deficient processing by Drosha. Alternative transcript splicing and polyadenylation can eliminate miRNA regulatory sites from a message, and miRNA directed repression can be blocked by certain RNA binding proteins. It seems less likely that common promoter element(s) or a single pathway (by itself) could explain the very large number of up- or down-regulated miRNAs noted as a result of oxygen restriction (Figure 1). Moreover, translation of miRNA targets leads to secondary transcriptional and post-transcriptional regulation that contributes to the observed mRNA profile. The diversity of potential regulatory sequences, difficulty predicting biologic regulation based solely on a consensus miRNA binding site, and the increasingly apparent need for confirmation in living cells indicate that additional, cell-based studies should be used in the future to address transcriptome regulation by miRNA.

miRNA expression arrays represent a relatively new technology, and potential issues exist with regard to data acquisition. The correlation of biological replicates in our studies was >0.99, which indicates the technology is precise, although accuracy is undefined. In addition, we randomized the order of miRNA and mRNA extraction to minimize non-biological, confounding variables. The goal of identifing a method to predict levels of mRNAs based on miRNA profiling, regardless of the underlying regulatory mechanism, was strengthened by correlation against predicted mRNA targets across the entire transcriptome. While previous studies have evaluated effects of a single miRNA after high level recombinant overexpression, the present experiments allowed us to study the dynamics of miRNA and mRNA regulation in parallel with a common physiologic insult (oxygen deprivation). This approach avoided potential variables introduced by overexpression of foreign DNA elements or otherwise manipulating the cellular genome.

The present findings suggest that correlation between miRNAs and their predicted targets based primarily on the number of consensus sites in the 3'UTR may be overly simplistic. Combinatorial analysis reveals much more significant agreement between specific genes and their predicted miRNA regulators as a group; however, this too may reflect a one-dimensional view of miRNA activity. Based on evidence presented here that entire (GO) functional categories of mRNAs are regulated in parallel by hypoxia (Table 2), higher order miRNA groupings may exist along functional or developmental lines that respond as networks. In either case, the present experiments provide a means by which other predicted target lists – either currently available or under development – may be optimized to yield a better correlation between miRNA levels and gene expression.

The observation that a gene-specific group of miRNAs may work in concert to repress CFTR mRNA during hypoxia also points to a novel mechanism of regulation. Previous experiments have failed to establish a direct role for HIF during the pronounced inhibition of CFTR that occurs during oxygen deprivation. Moreover, very few gene products are believed to be down-regulated in a direct fashion by HIF. If a cohort of miRNAs target CFTR and coordinately suppress its message, this could represent an important and novel example of miRNA based repression following an environmental stress. The findings may also help explain *in vivo *suppression of CFTR mRNA during low oxygen exposure[45,46], and suggest a role for miRNAs governing levels of hundreds of gene products following hypoxic insult (Figures 2 and 8).

## Conclusion

Our results suggest that the expected inverse relationship between miRNA and target mRNA may be a rare event. Several previous studies [13-15] have indicated a clear correlation between a specific miRNA and suppression of a target mRNA. These earlier studies in some cases were based on marked overexpression of a particular miRNA, followed by expression studies of the mRNAs of interest. However, our experiments suggest that under physiological conditions in human epithelium, miRNA acts in a more subtle fashion distinct from that of marked overexpression. In addition, the physiologic impact of miRNAs on cellular transcription appears to result from a multifaceted network of miRNA and mRNA relationships, working together in an interconnected system and in context of hundreds of other RNA species. It may be that target prediction algorithms and expression profiling techniques do not yet adequately represent the complexity of miRNA-mediated gene repression, and new methods may be required to truly understand these systemic aspects.

## Competing interests

The authors declare that they have no competing interests.

## Authors' contributions

JSG and JSH performed the experimental steps, data acquisition, and wrote the manuscript. HW assisted the experimental setup. SWE, TM, and GPP conducted data analysis. EJS supervised the project. All authors read and approved the final manuscript.

## Pre-publication history

The pre-publication history for this paper can be accessed here:


